# Definition and reliability of 3D acetabular and global offset measurements from bi-plane X-rays

**DOI:** 10.1038/s41598-023-27652-x

**Published:** 2023-01-11

**Authors:** Xavier Gasparutto, Pauline Besonhe, Peter Luca DiGiovanni, Stéphane Armand, Didier Hannouche

**Affiliations:** 1 Kinesiology Laboratory, Geneva University Hospitals and University of Geneva, Gabrielle-Perret-Gentil 4, 1211 Geneva, Switzerland; 2 Division of Orthopaedic Surgery and Musculoskeletal Trauma Care, Surgery Department, Geneva University Hospitals and University of Geneva, Gabrielle-Perret-Gentil 4, 1211 Geneva, Switzerland

**Keywords:** Medical research, Orthopaedics

## Abstract

The importance of the global offset, the sum of femoral and acetabular offset, has been underlined in the literature as a key factor for the functional outcome of total hip arthroplasty (THA). However, the acetabular offset is not defined for bi-plane X-rays, a technology providing 3D measurements of the lower limb and commonly used for patients undergoing THA. The aim of this paper is to introduce a measurement method of the 3D acetabular offset with bi-plane X-rays. Our method combines the use of technical and anatomical coordinate systems. The most appropriate definition will be selected based on the best reliability and measurement error. The consequent reliability of the global offset was also assessed. Twenty-eight patients undergoing primary THA were selected retrospectively. Two operators performed three reconstructions for each patients before and after THA. Intraclass correlation (ICC) and smallest detectable change (SDC) were computed for intra-operator, inter-operator and test–retest conditions for all combinations of technical and anatomical coordinate systems. ICCs were good to excellent. One combination was more reliable than others with a moderate mean SDC of 6.3 mm (4.3–8.7 mm) for the acetabular offset and a moderate mean SDC of 6.2 mm (5.6–6.7 mm) for the global offset. This is similar to the reliability and mean SDC of the femoral offset (4.8 mm) approved for clinical use which indicates that this method of acetabular offset measurement is appropriate. This opens a research avenue to better understand the role of the acetabular offset on THA outcomes, which seems overlooked in the literature.

## Introduction

The importance of the global offset, the sum of femoral and acetabular offset, has been underlined in the literature as a key factor for the functional outcome of total hip arthroplasty (THA)^1^. The acetabular and global offsets are defined for standard X-rays and CT-scans but not for bi-plane X-rays, although this technology is commonly used before and after surgery for the planning and evaluation of patients undergoing THA. The latter technology is a low dose imaging system that provides 3D parameters of the spine and lower-limb based on manual identification of anatomical landmarks on two orthogonal X-ray images (frontal and sagittal view) that are synchronised and spatially calibrated (EOS imaging, Paris, France). The advantage of 3D parameters instead of standard 2D parameters was underlined for lower limb parameters^2^. Indeed, femoral offset measurements showed higher reliability with bi-plane X-rays than with standard X-rays since bi-plane X-rays are less sensitive to the position of the patient with respect to the source of radiation^2^, such as differences in hip flexion–extension and/or internal–external rotation. It seems reasonable to assume that acetabular offset measurements could likewise be more reliable with bi-plane X-rays which gives an additional reason to the definition of this offset with this technology.

The aim of this paper is to propose a measurement method of the 3D acetabular offset based on bi-plane X-rays that is consistent between pre- and post-surgery evaluation and selected based on the best reliability and measurement error. Since bi-plane X-rays provide 3D measurements, we will define three acetabular offsets: the medio-lateral offset, the antero-posterior offset, and the vertical offset. The medio-lateral offset correspond to the standard acetabular offset currently used in the literature. The consequent reliability of the well defined global offset was also assessed.

## Method

### Design of the study

Twenty-eight patients were randomly selected retrospectively from a database of 439 patients that underwent primary unilateral THA at the Geneva University Hospitals between 2016 and 2019 and that had a bi-plane X-ray between one week and one month before, and two months after surgery. Patients with end-stage primary osteoarthritis were included while patients with other pathologies such as severe hip dysplasia, post-traumatic arthritis or post-infection sequelae were excluded. Two operators performed three reconstructions at each session for each patient using a research version of the dedicated sterEOS software (EOS imaging, Paris, France). Details of the protocol and statistical method are outlined in a previous study^3^ and data is shared on the online repository Yareta^4^. This study was approved by the local ethics committee (CCER Geneva, Switzerland). Patient’s informed consent was obtained and protected by the Geneva Ahtroplasty Registry and all experiments were performed in accordance with relevant guidelines and regulations.

### Acetabular offset definition

Two definitions of the acetabular offset are used in the literature. The first one, used with CT-scan, is defined as the distance between the centre of the femoral head and the true floor of the acetabulum^5^ or, for standard X-rays, by the horizontal distance to the pelvic teardrop^6^. The second definition relates to Pauwels’ balance^7^ as measured by the distance between the centre of the acetabulum and the sagittal plane of the pelvis^8^. Since the true floor is not visible on the sagittal X-rays and because the second definition is directly linked to the biomechanics of the hip, this definition will be followed and generalised in 3D by defining the additional antero-posterior and vertical offsets.

### Rationale of the method

When processing bi-plane X-rays with the sterEOS software, several pelvic anatomical landmarks are available: the acetabula, the centre of the sacral slope, the pubic symphysis, and the anterior superior iliac spines. To compare pre- and post-surgery offsets, the anatomical planes of the pelvis should be identical before and after surgery. Between the pre- and post-THA bi-plane X-rays, the operated acetabulum is the only affected landmark so the others can be used. However, the antero-superior iliac spines position have poor reliability^3^, thus the anterior pelvic plane will not be used as a plane of reference. The available landmarks with good reliability therefore include the centre of the sacral slope, the pubic symphysis, and the contralateral acetabulum. Although these points do not define anatomical planes of the pelvis, they can be used to link the pre- and post-surgery measurements while the anatomical planes are defined only once, on pre-surgery X-rays. To that end, 3D cartesian coordinate systems will be used.

### Principle of the method

**Figure 1:**
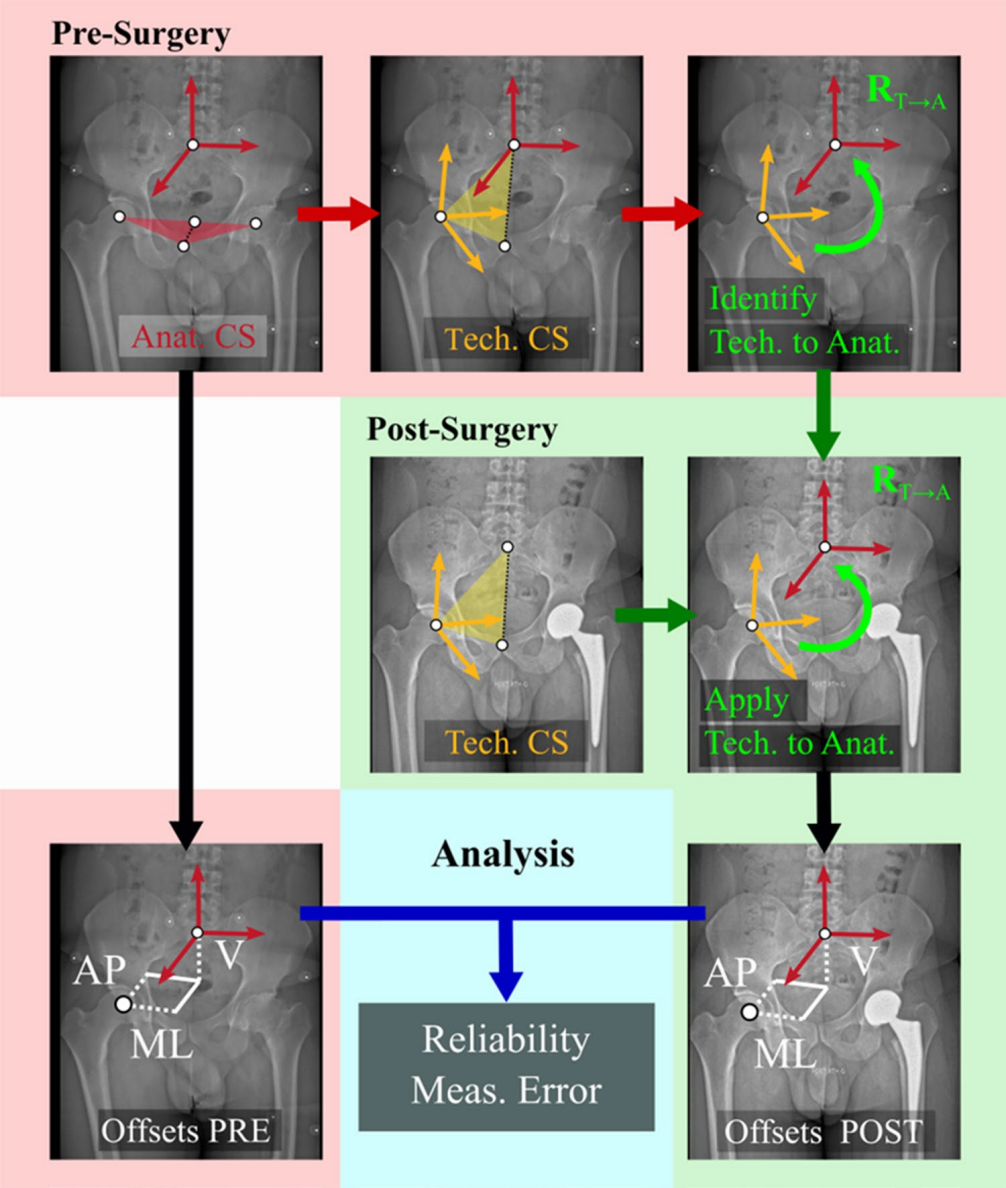
Workflow of the method. *Tech* stands for technical, *Anat* for anatomical, *CS* for coordinate system, *AP* for antero-posterior, *ML* for medio-lateral, *V* for vertical, and *Meas.* for measurement. ${{\varvec{R}}}_{T\to A}$ represents the orientation of the technical coordinate system with respect to the anatomical coordinate system.

Five steps are required to obtain similar anatomical coordinate systems on the pre- and post-surgery X-rays (Fig. 1). First, the anatomical coordinate system is defined on the pre-surgery X-rays. This coordinate system defines the anatomical planes of the pelvis and the measure of acetabular offsets. Second, the technical coordinate system is identified on the same pre-surgery X-rays. Third, the orientation of the technical coordinate system with respect to the anatomical one is identified. Next, the technical coordinate system is identified on the post-surgery X-rays. Lastly, the geometric transformation between the technical and anatomical coordinate systems is applied post-surgery, to obtain the same orientation of the anatomical coordinate system as pre-surgery on the post-surgery X-rays. The origin of this new coordinate system is set as the anatomical landmark specified by the definition and identified on the post-surgery X-rays.

Since the anatomical planes of the pelvis pre- and post-surgery are defined in the same way, it is possible to compute the acetabular offsets with the same definition of the anatomical plane, and thus to evaluate the change after surgery. The acetabular offsets will represent the distance of the centre of the acetabulum to the origin of the anatomical coordinate system expressed along the medio-lateral, antero-posterior, and vertical axes of the coordinate system. As these parameters are lengths, their values will be positive.

### Coordinate system definitions

**Figure 2:**
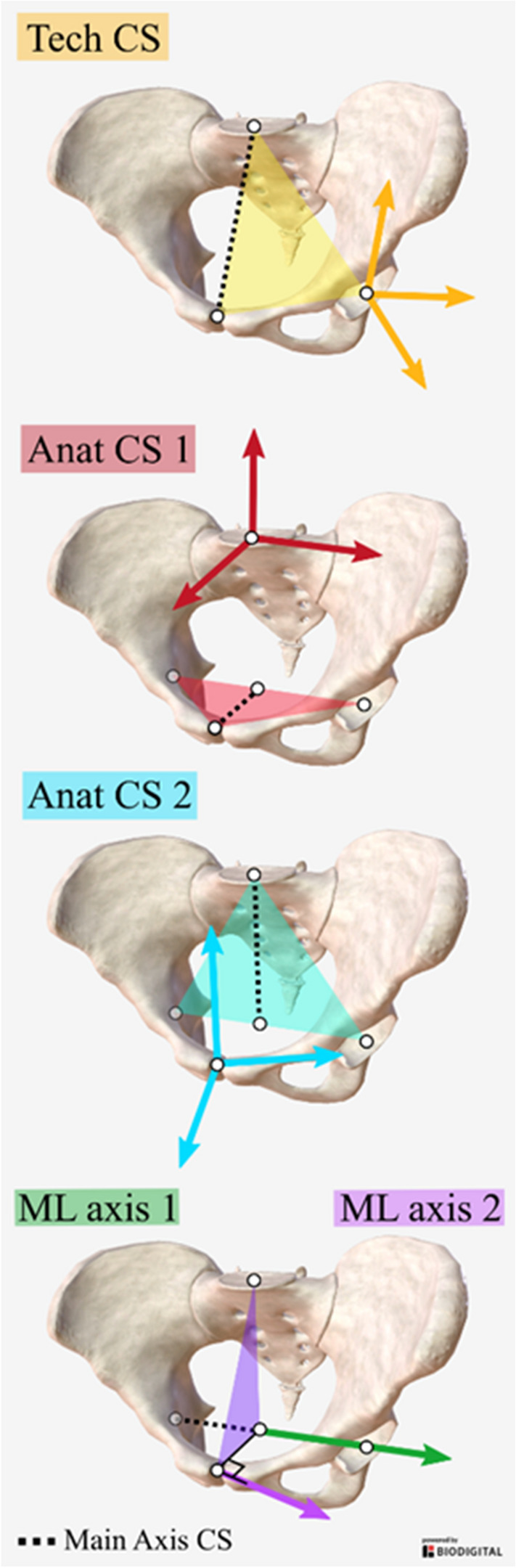
Definition of technical (Tech) and anatomical (Anat) coordinate systems (CS) and the two medio-lateral (ML) axis definition. Pelvis images were obtained from www.biodigital.com.

Multiple definitions of technical and anatomical coordinate systems are possible based on the anatomical landmarks available. We will first present the definition of the multiple coordinate systems (Fig. 2) and then the method to evaluate the reliability of the measurements.

### Technical coordinate system

The coordinate system has three axes and one origin. Three points are available, thus we can only define one set of three axes. Multiple origins are possible, but since the origin of the anatomical coordinate system post-surgery is the anatomical landmark measured post-surgery, the origin of the technical frame will not influence the values of the offsets. The origin of the technical coordinate system was arbitrarily chosen as the centre of the acetabulum. Details of the computations are presented in supplementary file 1.

### Anatomical coordinate systems

Two definitions are possible for the medio-lateral axis. The first describes the line from one acetabulum to the other (ML1). The second is the vector orthogonal to the plane defined by the midpoint of the acetabula, the pubic symphysis, and the centre of the plane slope (ML2).

Regarding the antero-posterior and vertical axis, two definitions are also possible. First, the antero-posterior axis is defined by the midpoints of the acetabulum and the pubic symphysis. The vertical axis will then be defined as the cross product of the antero-posterior and medio-lateral axes. For the second definition, the vertical axis is defined by the midpoint of the acetabula and the centre of the sacral slope. The antero-posterior axis will then be defined as the cross product of the medio-lateral and vertical axes. Details of computations are presented in supplementary file 1.

To avoid an offset value of zero, the origin of the anatomical coordinate system needs to be outside of the plane of the three points used to build the three axes. Thus, for the first anatomical coordinate system, the origin can only be the centre of the sacral slope and for the second anatomical coordinate system, the origin can only be the pubic symphysis.

By combining the different axis definitions, four possibilities exist for the anatomical coordinate systems.

### Evaluation of the method

The acetabular offsets of the contralateral acetabulum are not impacted by the surgery, thus it is possible to evaluate the intra-operator, inter-operator and test–retest reliability with this parameter. According to the COSMIN taxonomy and checklist^9^, the reliability domain contains the reliability, assessed with intraclass correlation coefficient (ICC), and the measurement error, assessed with the smallest detectable change (SDC). The ICCs were classified as follow: poor (< 0.5), moderate (0.5–0.75), good (0.75–0.9), and excellent (> 0.9)^10^. The SDCs were classified as follow: poor (> 10 mm/°), moderate (5 to 10 mm/°), good (3 to 5 mm/°), and excellent (< 3 mm/°)^3^.

The reliability of the offsets computed with the twelve combinations of technical and anatomical coordinate systems was assessed. The combination with the highest reliability of acetabular and global offset was chosen as the best definition. The target for an acceptable measurement was to achieve similar or better ICC and SDC as the femoral offset approved for clinical use. These reliability outcomes of the femoral offset were evaluated with the same database in a previous study^3^ and resulted in a good to excellent ICC (0.853 to 0.916) and an average SDC of 4.8 mm (4.3–5.7 mm).

The mean values of the offsets are reported in Table 1. All ICCs were rated as good to excellent for acetabular offsets (Table 2) and global offset (Table 3). The anatomical coordinate system defined by the acetabula and centre of the sacral slope (A2) with the medio-lateral axis defined by the acetabula (ML1) had lower SDCs than other acetabular offsets and presented a mean SDC over the three-axis of 6.3 mm (4.3 mm to 8.7 mm) (Table 2). For this definition, the average SDC was equal to 13% of the offsets values—13.5%, 5.3%, and 26.1% for the antero-posterior, medio-lateral and vertical directions, respectively. The mean SDCs of the global offset were all moderate (6.3–8.8 mm, Table 3) and represented 5.2% (4.7–6.7%) of the mean offset value.

**Table 1:** Mean (SD) values of the offsets (in mm).

ACS	Medio-Lat Axis ACS	Origin ACS	Ant-post Mean (SD)	Medio-Lat Mean (SD)	Vertical Mean (SD)	Global Mean (SD)
A1	ML1	SSC	53.2 (13.5)	89.6 (7.1)	85.5 (14.2)	131.9 (10.3)
A2	ML1	PSYM	47.5 (8.2)	88.8 (5.8)	29.6 (8.2)	131.2 (9.3)
A2	ML2	PSYM	44.4 (12.8)	89.6 (6.1)	30 (9.6)	131.9 (9.5)
A1	ML2	SSC	55 (15.1)	89.6 (6.1)	83.3 (18.5)	131.9 (9.5)

TCS: Technical Coordinate System; ACS: Anatomical Coordinate System; T1,2,3: technical coordinate systems; A1,2,3: Anatomical coordinate systems; ML1,2: definitions of the medio-lateral axis; SSC: sacral slope centre; PSYM: pubic symphysis; ACET: acetabulum.

**Table 2:**
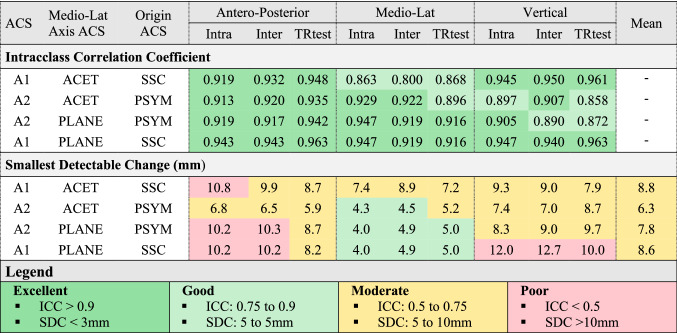
Intraclass correlation coefficient and smallest detectable change (in mm) of the three acetabular offsets for all combinations of technical and anatomical coordinate systems.

The ACET convention uses acetabula to define the medio-lateral axis. The PLANE convention use the normal to the plane defined by the centre of the sacral slope (SSC), the pubic symphisys (PSYM) and the midpoint of the acetabula to define the medio-lateral axis.

**Table 3:**
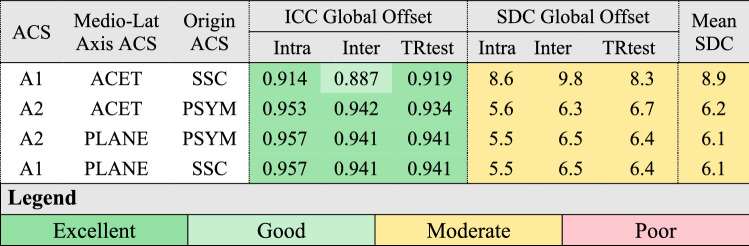
Intraclass correlation (ICC) coefficient and smallest detectable change (SDC, in mm) of the global offset for all combinations of technical and anatomical coordinate systems.

The ACET convention uses acetabula to define the medio-lateral axis. The PLANE convention use the normal to the plane defined by the centre of the sacral slope (SSC), the pubic symphisys (PSYM) and the midpoint of the acetabula to define the medio-lateral axis.

## Discussion

This study achieved the goal of defining a measurement method of 3D acetabular offsets consistent between pre- and post-surgery evaluation with bi-plane X-rays. The combination of the anatomical coordinate system defined by the acetabula and centre of the sacral slope (A2) with the medio-lateral axis defined by the acetabula (ML1) reached the same reliability, despite slightly higher measurement error, as the femoral offset approved for clinical use for both acetabular and global offsets. Indeed, the femoral offset presented ICCs between 0.853 and 0.916 and a mean SDC of 4.8 mm with the same dataset^3^ while, with the selected definition, the ICC were between 0.858 and 0.953 with mean SDCs of 6.3 mm and 6.2 mm for the acetabular and global offsets, respectively.

For the global offset, this SDC represents only 5.2% of its mean value and all ICCs are excellent which underlines the relevance of the present definition. Regarding the acetabular offset, although the SDCs represent up to 26.1% of the vertical acetabular offset, the intra-operator, inter-operator and test-retests ICCs were all good to excellent. Thus, the present definition was suitable to differentiate patients and to evaluate pre- post-surgery changes of patients’ acetabular offsets. It is noticeable that the two definitions of anatomical coordinate systems gave the same results for the medio-lateral offset when using the second definition of medio-lateral axis, although the origins of these coordinate systems are different. This axis is defined as the norm to the plane defined by the pubic symphysis, the centre of the sacral slope and the midpoint of the acetabulums. Thus, by definition, the distance from the contralateral acetabulum to the pubic symphysis and to the sacral slope centre along this axis will be the distance to the plane and will be identical.

The relevance and choice of the definition of the anatomical plane could have been done by a consensus among clinicians or anatomists since both definitions will provide different values of offsets. However, for the clinician, the change in offset between pre- and post-surgery is of greater importance than the absolute value at a timepoint. This understanding of the impact of the change in offsets is widely covered in the literature^1,8,11^. Thus, since both definitions seemed anatomically relevant and since the assessment of change requires the highest possible reliability, the choice was made to use the reliability and measurement error as criteria to have the best possible measurement with this specific technology. In this way, the definition takes into account the characteristics and anatomical landmarks provided by the medical imaging technique, i.e. bi-plane X-rays. Still, the present method could be applied directly to other 3D imaging techniques such as CT-scan and could also be applied to other geometrical parameters.

Regarding the reasons why this specific definitions has a better SDC than the others, the authors identified three hypothesis. First, the definitions using the pubic symphysis as origin of the LCS had better measurement error than the definitions using the centre of the sacral slope. This seems like a natural results since the pubic symphysis has a better measurement error than the centre of the sacral slope with bi-plane X-rays^3^. Second, in this definition the centre of the sacral slope, the point with the lowest reliability^3^, is used only to define the vertical axis, and will thus have only a slight impact on the pelvic tilt of the pelvic LCS which, in turn, will have no impact on the nedio-lateral offset, and only a low impact on the vertical and anterior–posterior offset. Third, the acetabula, used in the 1st definition of the medio-lateral axis, have a higher reliability than the centre of the sacral slope used in the 2nd definition of the medio-lateral axis, which could explain the better reliability of the 1st definition.

This 3D approach of evaluating the acetabular offset contrasts with the standard 2D approach. This 3D approach could prove more reliable than the 2D approach for the medial–lateral and vertical acetabular offset as shown for lower limb measurements^2^. The 3D approach also introduces the anterior–posterior acetabular offset which, to our knowledge, has not been previously studied. Moreover, 2D approaches use the radiological plane as the frontal plane of the patient such as in Mahmood et al.^1^ for the definition of femoral and acetabular offset. This assumption is reasonable but dependent on patient posture, especially axial rotation. On the contrary, the present approach defines anatomical planes independently of the patient’s posture by using coordinate systems linked to the pelvis. The test–retest SDC were similar to the inter- and intra-operator SDC, which indicates that patient positioning has low influence on the reliability of the measurement.

The SDC of the antero-posterior offset was higher than the medio-lateral and vertical offsets. This was expected since a previous study using the same database showed that the sagittal X-ray had lower reliability than the frontal plane X-ray^3^.

The reliability and measurement errors of the global offset were similar for three different definitions of the acetabular offset. Thus, among those three definitions, the final selection of the acetabular offset definition was based only on the the best reliability of the acetabular offset.

The current study does have some limitations. The number of patients could have been increased to achieve higher statistical power and more than two operators could be included as the number of operators is frequently larger in a radiological department. On the methodological side, pre-surgery X-rays are needed to define the offsets on the post-surgery X-rays and, to compute offsets, the positions of anatomical landmarks need to be exported which is not standard with the sterEOS software. A slightly more time-consuming workaround can be performed by using the built-in 3D tools to add specific anatomical landmarks that can be exported with the clinical version of the software.

## Conclusion

This study defined a 3D acetabular offset with similar levels of reliability and measurement error as the femoral offset approved for clinical use, i.e. a good to excellent ICC and a good to moderate SDC. The present method can be used to evaluate the change of the 3D offset before and after THA by keeping the same definition of anatomical planes at both visits. This opens a research avenue to better understand the role of the acetabular offset on THA outcomes, which seems overlooked in the literature.

## Supplementary Information

Supplementary Information.

## Data Availability

Data is available on Yareta: https://doi.org/10.26037/yareta:2al7pd6y7rgkzkd6nolwp6ucji.
