# Supplementary material for: Definition and reliability of 3D acetabular and global offset measurements from bi-plane X-rays
Source: Sci Rep. 2023 Jan 11;13:591. doi: 10.1038/s41598-023-27652-x (PMC9834380; doi:10.1038/s41598-023-27652-x)
Supplement: Supplementary file 1 — Supplementary Information. [file 41598_2023_27652_MOESM1_ESM.docx]

Computation of the coordinate systems

| 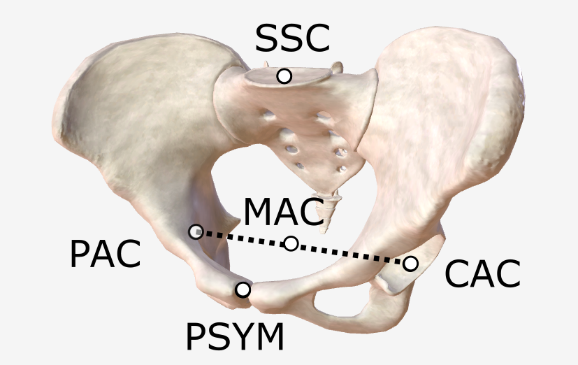 | **Acronym** | **Anatomical Points** |
| --- | --- | --- |
|  | **SSC** | Centre of the sacral slope |
|  | **CAC** | Contralateral Acetabulum (Left for example) |
|  | **PAC** | Pathological Acetabulum (Right for example) |
|  | **MAC** | Midpoint of the right and left acetabulum |
|  | **PSYM** | Pubic Symphysis |

In the following definition, the acronyms of the anatomical will define the position of the points expressed in the coordinate system reported as index. As an example, $\boldsymbol{CAC}_{\boldsymbol{e}}$ represents the position of the contralateral acetabulum measured in the coordinate system of the bi-plane X-ray, as measured by the operator on the image.

# Technical Coordinate System

Origin: $\boldsymbol{O}_{t/e}=\boldsymbol{CAC}_{\boldsymbol{e}}$;

Where the index *e* denotes that the position is expressed in the coordinate system of the bi-plane image.

### Axis definition:

$$\boldsymbol{Z}_{e\to t}=\frac{\boldsymbol{SSC}_{\boldsymbol{e}}-\boldsymbol{PSYM}_{\boldsymbol{e}}}{\left\| \boldsymbol{SSC}_{\boldsymbol{e}}-\boldsymbol{PSYM}_{\boldsymbol{e}} \right\|}$$

$$\boldsymbol{Y}_{tmp}=\boldsymbol{PSYM}-\boldsymbol{CAC}$$

$$\boldsymbol{X}_{e\to t}=\frac{\boldsymbol{Y}_{tmp}\times\boldsymbol{Z}_{e\to t}}{\left\| \boldsymbol{Y}_{tmp}\times\boldsymbol{Z}_{e\to t} \right\|}$$

$$\boldsymbol{Y}_{e\to t}=\frac{\boldsymbol{Z}_{e\to t}\times\boldsymbol{X}_{e\to t}}{\left\| \boldsymbol{Z}_{e\to t}\times\boldsymbol{X}_{e\to t} \right\|}$$

Rotation matrix : $\boldsymbol{R}_{e\to t}=[\begin{matrix} \boldsymbol{X}_{e\to t} & \boldsymbol{Y}_{e\to t} & \boldsymbol{Z}_{e\to t} \end{matrix} ]$

Transformation matrix : $\boldsymbol{T}_{g\to t}=\left[ \begin{matrix} \boldsymbol{R}_{g\to t} & \boldsymbol{O}_{t/g} \\ 0 0 0 & 1 \end{matrix} \right]$

Where $e\to t$ denotes that the vector is used to define the axis of the technical LCS (*t*) in the coordinate system of the bi-plane image (*e*) and $\times$ denotes the cross-product between two vectors.

# Medio-lateral axis

## Definition 1: **ML axis 1**

$$\boldsymbol{ML}_{e\to p}^{\boldsymbol{1}}=\frac{\boldsymbol{CAC}_{\boldsymbol{e}}-\boldsymbol{PAC}_{\boldsymbol{e}}}{\left\| \boldsymbol{CAC}_{\boldsymbol{e}}-\boldsymbol{PAC}_{\boldsymbol{e}} \right\|}$$

Where $e\to p$ denotes that the vector is used to define the medio-lateral axis of the pelvis (*p*) in the coordinate system of the bi-plane image (*e*).

## Definition 2: **ML axis 2**

$$\boldsymbol{Z}_{tmp}=\boldsymbol{SSC}_{\boldsymbol{e}}-\boldsymbol{MAC}_{\boldsymbol{e}}$$

$$\boldsymbol{Y}_{tmp}=\boldsymbol{PSYM}_{\boldsymbol{e}}-\boldsymbol{MAC}_{\boldsymbol{e}}$$

$$\boldsymbol{ML}_{e\to p}^{\boldsymbol{2}}=\frac{\boldsymbol{Y}_{tmp}\times\boldsymbol{Z}_{tmp}}{\left\| \boldsymbol{Y}_{tmp}\times\boldsymbol{Z}_{tmp} \right\|}$$

# Anatomical coordinate systems

## Definition 1: **Anat CS1**

Origin : $\boldsymbol{O}_{p/e}^{\boldsymbol{1}}=\boldsymbol{SSC}_{\boldsymbol{e}}$

### Axis definition :

$$\boldsymbol{X}_{tmp}=\boldsymbol{PSYM}_{\boldsymbol{e}}-\boldsymbol{MAC}_{\boldsymbol{e}}$$

$$\boldsymbol{Z}_{e\to p}^{\boldsymbol{1}}=\frac{\boldsymbol{X}_{tmp}\times\boldsymbol{ML}_{e\to p}}{\left\| \boldsymbol{X}_{tmp}\times\boldsymbol{ML}_{e\to p} \right\|}$$

$$\boldsymbol{X}_{e\to p}^{\boldsymbol{1}}=\frac{\boldsymbol{Y}_{e\to p}\times\boldsymbol{Z}_{e\to p}}{\left\| \boldsymbol{Y}_{e\to p}\times\boldsymbol{Z}_{e\to p} \right\|}$$

Rotation matrix : $\boldsymbol{R}_{e\to p}^{\boldsymbol{1}}=[\begin{matrix} \boldsymbol{X}_{e\to p}^{\boldsymbol{1}} & \boldsymbol{ML}_{e\to p} & \boldsymbol{Z}_{e\to p}^{\boldsymbol{1}} \end{matrix} ]$

Transformation matrix : $\boldsymbol{T}_{e\to p}^{\boldsymbol{1}}=\left[ \begin{matrix} \boldsymbol{R}_{e\to p}^{\boldsymbol{1}} & \boldsymbol{O}_{p/e}^{\boldsymbol{1}} \\ 0 0 0 & 1 \end{matrix} \right]$

Where $\boldsymbol{ML}_{e\to p}$ can be either $\boldsymbol{ML}_{e\to p}^{\boldsymbol{1}}$ or $\boldsymbol{ML}_{e\to p}^{\boldsymbol{2}}$ depending on the chosen convention.

## Definition 2: **Anat CS2**

Origin : $\boldsymbol{O}_{t/p}=\boldsymbol{PSYM}_{\boldsymbol{e}}$

### Axis definition :

$$\boldsymbol{Z}_{tmp}=\boldsymbol{SSC}-\boldsymbol{MAC}$$

$$\boldsymbol{X}_{e\to p}^{\boldsymbol{2}}=\frac{\boldsymbol{ML}_{e\to p}\times\boldsymbol{Z}_{tmp}}{\left\| \boldsymbol{ML}_{e\to p}\times\boldsymbol{Z}_{tmp} \right\|}$$

$$\boldsymbol{Z}_{e\to p}^{\boldsymbol{2}}=\frac{\boldsymbol{X}_{e\to p}\times\boldsymbol{Y}_{e\to p}}{\left\| \boldsymbol{X}_{e\to p}\times\boldsymbol{Y}_{e\to p} \right\|}$$

Rotation matrix : $\boldsymbol{R}_{e\to p}^{\boldsymbol{2}}=[\begin{matrix} \boldsymbol{X}_{e\to p}^{\boldsymbol{2}} & \boldsymbol{ML}_{e\to p} & \boldsymbol{Z}_{e\to p}^{\boldsymbol{2}} \end{matrix} ]$

Transformation matrix : $\boldsymbol{T}_{e\to p}^{\boldsymbol{2}}=\left[ \begin{matrix} \boldsymbol{R}_{e\to p}^{\boldsymbol{2}} & \boldsymbol{O}_{p/e}^{\boldsymbol{2}} \\ 0 0 0 & 1 \end{matrix} \right]$

Where $\boldsymbol{ML}_{e\to p}$ can be either $\boldsymbol{ML}_{e\to p}^{\boldsymbol{1}}$ or $\boldsymbol{ML}_{e\to p}^{\boldsymbol{2}}$ depending on the chosen convention.

Computation of acetabular offset

# Pre-surgery

1. Compute the pelvis coordinate system $\boldsymbol{T}_{e\to p}^{\boldsymbol{pre}}$ – see previous section
2. Express the centre of the acetabulum measured on the pre-surgery images in the pre-surgery pelvis coordinate system:

$\left[ \begin{matrix} \boldsymbol{AC}_{\boldsymbol{p}}^{\boldsymbol{pre}} \\ \boldsymbol{1} \end{matrix} \right]\boldsymbol{=}\left( \boldsymbol{T}_{e\to p}^{\boldsymbol{pre}} \right)^{\boldsymbol{-1}}\boldsymbol{.}\left[ \begin{matrix} \boldsymbol{AC}_{\boldsymbol{e}}^{\boldsymbol{pre}} \\ \boldsymbol{1} \end{matrix} \right]$ with $\boldsymbol{AC}_{\boldsymbol{p}}^{\boldsymbol{pre}}\boldsymbol{=}\left[ \begin{matrix} \boldsymbol{X}_{\boldsymbol{AC}_{\boldsymbol{p}}^{\boldsymbol{pre}}} \\ \boldsymbol{Y}_{\boldsymbol{AC}_{\boldsymbol{p}}^{\boldsymbol{pre}}} \\ \boldsymbol{Z}_{\boldsymbol{AC}_{\boldsymbol{p}}^{\boldsymbol{pre}}} \end{matrix} \right]$

1. Since offsets are distances, the acetabular offsets are the absolute values of the coordinates of the centre of the acetabulum expressed in the pre-surgery pelvis coordinate system, i.e $\boldsymbol{AC}_{\boldsymbol{e}}^{\boldsymbol{pre}}$:

| **Coordinate** | **Acetabular offset** |
| --- | --- |
| $abs\boldsymbol{(}\boldsymbol{X}_{\boldsymbol{AC}_{\boldsymbol{p}}^{\boldsymbol{pre}}}\boldsymbol{)}$ | Anterior-posterior offset |
| $abs\boldsymbol{(}\boldsymbol{Y}_{\boldsymbol{AC}_{\boldsymbol{p}}^{\boldsymbol{pre}}}\boldsymbol{)}$ | Medio-lateral offset |
| $abs\boldsymbol{(}\boldsymbol{Z}_{\boldsymbol{AC}_{\boldsymbol{p}}^{\boldsymbol{pre}}}\boldsymbol{)}$ | Vertical offset |

1. Compute the technical coordinate system $\boldsymbol{T}_{g\to t}^{\boldsymbol{pre}}$ – see previous section
2. Compute the transformation between the technical coordinate system and the pelvis coordinate system as follow:

$$\boldsymbol{T}_{t\to p}^{\boldsymbol{pre}}=\left( \boldsymbol{T}_{g\to t}^{\boldsymbol{pre}} \right)^{-1}.\boldsymbol{T}_{g\to p}^{\boldsymbol{pre}}$$

This transformation matrix will be used in the next section

# Post-surgery

1. Compute the technical coordinate system $\boldsymbol{T}_{e\to t}^{\boldsymbol{post}}$– see previous section
2. Apply the transformation between the technical coordinate system measured post-surgery and pelvis coordinate system defined pre-surgery to define the pelvic coordinate system post-surgery:

$$\boldsymbol{T}_{e\to p}^{\boldsymbol{post}}=\boldsymbol{T}_{g\to t}^{\boldsymbol{post}}.\boldsymbol{T}_{t\to p}^{\boldsymbol{pre}}$$

3. Express the centre of the acetabulum measured post-surgery in the post-surgery pelvis coordinate system:

$\left[ \begin{matrix} \boldsymbol{AC}_{\boldsymbol{p}}^{\boldsymbol{post}} \\ \boldsymbol{1} \end{matrix} \right]\boldsymbol{=}\left( \boldsymbol{T}_{e\to p}^{\boldsymbol{post}} \right)^{\boldsymbol{-1}}\boldsymbol{.}\left[ \begin{matrix} \boldsymbol{AC}_{\boldsymbol{e}}^{\boldsymbol{post}} \\ \boldsymbol{1} \end{matrix} \right]$ with $\boldsymbol{AC}_{\boldsymbol{p}}^{\boldsymbol{post}}\boldsymbol{=}\left[ \begin{matrix} \boldsymbol{X}_{\boldsymbol{AC}_{\boldsymbol{p}}^{\boldsymbol{post}}} \\ \boldsymbol{Y}_{\boldsymbol{AC}_{\boldsymbol{p}}^{\boldsymbol{post}}} \\ \boldsymbol{Z}_{\boldsymbol{AC}_{\boldsymbol{p}}^{\boldsymbol{post}}} \end{matrix} \right]$

1. Since offsets are distances, the acetabular offsets are the absolute values of the coordinates of the centre of the acetabulum expressed in the post-surgery pelvis coordinate system, i.e $\boldsymbol{AC}_{\boldsymbol{e}}^{\boldsymbol{post}}$:

| **Coordinate** | **Acetabular offset** |
| --- | --- |
| $abs\boldsymbol{(}\boldsymbol{X}_{\boldsymbol{AC}_{\boldsymbol{p}}^{\boldsymbol{post}}}\boldsymbol{)}$ | Anterior-posterior offset |
| $abs\boldsymbol{(}\boldsymbol{Y}_{\boldsymbol{AC}_{\boldsymbol{p}}^{\boldsymbol{post}}}\boldsymbol{)}$ | Medio-lateral offset |
| $abs\boldsymbol{(}\boldsymbol{Z}_{\boldsymbol{AC}_{\boldsymbol{p}}^{\boldsymbol{post}}}\boldsymbol{)}$ | Vertical offset |
